# RNA recognition motifs of disease-linked RNA-binding proteins contribute to amyloid formation

**DOI:** 10.1038/s41598-019-42367-8

**Published:** 2019-04-16

**Authors:** Sashank Agrawal, Pan-Hsien Kuo, Lee-Ya Chu, Bagher Golzarroshan, Monika Jain, Hanna S. Yuan

**Affiliations:** 1 Molecular and Cell Biology, Taiwan International Graduate Program, Academia Sinica and Graduate Institute of Life Sciences, National Defense Medical Center, Taipei, Taiwan; 2 Institute of Molecular Biology, Academia Sinica, Taipei, Taiwan; 3 Chemical Biology and Molecular Biophysics, Taiwan International Graduate Program, Academia Sinica, Taipei, Taiwan; 4 Institute of Bioinformatics and Structural Biology, National Tsing Hua University, Hsin Chu, Taiwan

## Abstract

Aberrant expression, dysfunction and particularly aggregation of a group of RNA-binding proteins, including TDP-43, FUS and RBM45, are associated with neurological disorders. These three disease-linked RNA-binding proteins all contain at least one RNA recognition motif (RRM). However, it is not clear if these RRMs contribute to their aggregation-prone character. Here, we compare the biophysical and fibril formation properties of five RRMs from disease-linked RNA-binding proteins and five RRMs from non-disease-associated proteins to determine if disease-linked RRMs share specific features making them prone to self-assembly. We found that most of the disease-linked RRMs exhibit reversible thermal unfolding and refolding, and have a slightly lower average thermal melting point compared to that of normal RRMs. The full domain of TDP-43 RRM1 and FUS RRM, as well as the β-peptides from these two RRMs, could self-assemble into fibril-like aggregates which are amyloids of parallel β-sheets as verified by X-ray diffraction and FT-IR spectroscopy. Our results suggest that some disease-linked RRMs indeed play important roles in amyloid formation and shed light on why RNA-binding proteins with RRMs are frequently identified in the cellular inclusions of neurodegenerative diseases.

## Introduction

RNA-binding proteins play primary roles in RNA metabolism, coordinating networks of RNA-protein and protein-protein interactions and regulating many important events such as RNA splicing, maturation, translation, transport and turnover. Aberrant expression, dysfunction, and aggregation of RNA-binding proteins have been identified in several major classes of human diseases, including neurological disorders, muscular atrophies and cancer^1,2^. In particular, aggregation of RNA-binding proteins is considered to be one of the major hallmarks of neurodegenerative diseases, based on characterization of cellular inclusions containing TAR DNA-binding protein 43 (TDP-43) and Fused in Sarcoma (FUS) in the diseased neurons of patients suffering amyotrophic lateral sclerosis (ALS) and fronto-temporal lobar dementia (FTLD)^3–7^. RNA-binding proteins usually contain one or more RNA recognition motifs (RRMs), raising the intriguing question as to whether RRMs can mediate protein aggregation.

TDP-43 is the key component of toxic cytosolic inclusions in ALS and FTLD^8^. TDP-43 contains an N-terminal domain (NTD), two RRMs (RRM1 and RRM2), and an unstructured C-terminal domain (CTD)^9^. Most ALS- and FTLD-linked mutations are located within the CTD, so its unstructured Glycine-rich region has been extensively studied for its involvement in protein aggregation^10–12^. A prion-like region has been identified in the CTD, and small peptides in the Glycine-rich region form twisted fibrils *in vitro*^13–20^. However, the major components in the cytoplasmic inclusions of diseased neurons in ALS/FTLD patients are 35-kD and 25-kD C-terminal fragments of TDP-43, comprising not only the CTD but also RRM1 and RRM2^21–23^. Some studies have demonstrated that RRMs or parts thereof could be involved in TDP-43 aggregation and cytotoxicity. For example, expression of a series of TDP-43 truncation fragments showed that the CTD alone is not efficient at inducing protein aggregation and that RRMs are required for robust protein aggregation in the cytoplasm of mammalian^24–26^ and yeast cells^27^. Overexpression of RRM1-deleted TDP-43 in a transgenic *Drosophila* model also demonstrated that RRM1 deletion mitigated the degeneration of motor neurons compared to the effect of full-length TDP-43^28^. Moreover, our previous study revealed that two small peptides in the β strand region of TDP-43 RRM2—β3 (residues 227–233) and β5 (residues 253–259)—as well as a truncated RRM2 (residues 208–265), can form fibril-like aggregates *in vitro*^26^. Recently, it has also been reported that RRMs from TDP-43 have amyloid-like properties^29,30^ and that various peptides from the TDP-43 RRM2 domain can form amyloid fibrils *in vitro*^31,32^, further supporting the role of RRMs in promoting protein aggregation and amyloid formation.

Similar to TDP-43, the RNA-binding proteins FUS and RNA-binding motif 45 (RBM45) have also been identified in the cytoplasmic inclusions of ALS and FTLD patients^5,7,33,34^. As for TDP-43, mislocalization and inclusions of FUS are not limited to ALS and FTLD but have also been detected in other neurodegenerative diseases, including Huntington disease^35–37^. FUS contains a QGSY-rich prion-like domain, a Glycine-rich region, a C-terminal low complexity domain and a single RRM, with this RRM necessary for manifesting FUS cytotoxicity^38^ and being capable of self-assembling into amyloid fibrils *in vitro*^39^. In contrast, RBM45 only comprises three RRMs (RRM1-3), thus lacking any other structural domain, but the role of RRMs in RBM45 aggregation is not clear. RBM45 forms homo-oligomers and physically associates with TDP-43 and FUS in the nucleus^40^, suggesting a close link between RBM45, TDP-43 and FUS in aberrant RNA metabolism and protein aggregation in ALS.

**Figure 1:**
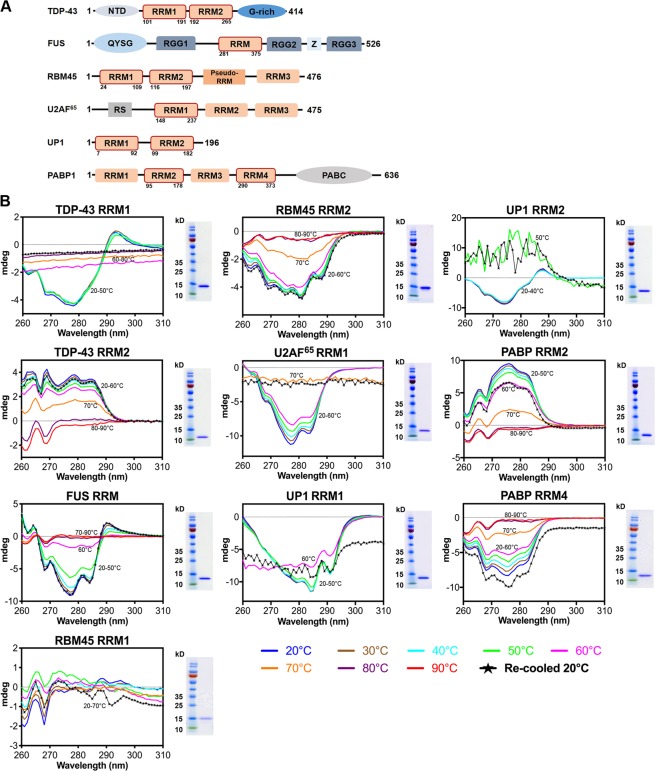
Most disease-linked RNA recognition motifs (RRMs) exhibit reversible thermal unfolding and refolding. (**A**) Domain organization of six RNA-binding proteins shows the ten RRMs (outlined in red box with residue numbers marked at the bottom) used in this study. (**B**) Overall tertiary structural changes for five RRMs from disease-linked RNA-binding proteins (TDP-43 RRM1, TDP-43 RRM2, FUS RRM, RBM45 RRM1, RBM45 RRM2), and five RRMs from non-disease-associated proteins (U2AF^47^ RRM1, UP1 RRM1, UP1 RRM2, PABP RRM2, PABP RRM4), assessed by circular dichroism in the near-UV range (260 to 310 nm) during the thermal unfolding and refolding process. We raised the temperature from 20 °C to 90 °C at intervals of 10 °C (marked by different colors, as shown at the bottom of the figure) to induce protein unfolding, and then re-cooled to 20 °C for protein refolding (marked by lines of black asterisks, labeled as “Re-cooled 20 °C”). Each RRM was purified to a high homogeneity, as shown by the SDS-PAGE gels at right of the CD spectra (The full-length gels are shown in the Supplementary Fig. S1).

In fact, RRMs are the only domains shared by these three aggregation-prone RNA-binding proteins, TDP-43, FUS and RBM45 (see Fig. 1A). RRMs are RNA-binding domains of 80–90 amino acids that contain two highly conserved consensus sequences (RNP1 and RNP2 of 8 and 6 residues, respectively)^41^. RRMs are folded, with two α-helices packed against a β-sheet consisting of four or five antiparallel β-strands^42^. The crystal and NMR structures of RRM1 and RRM2 of TDP-43 reveal how these two RRMs bind nucleic acids^43–45^. Here, we compared the biophysical and fibril-formation properties of five RRMs from the disease-linked proteins, TDP-43, FUS and RBM45, as well as five RRMs from RNA-binding proteins not linked to disease, i.e., U2AF^46,47^, UP1^48^, and PABP^49^. We observed that most of the disease-linked RRMs display reversible thermal unfolding-refolding properties and have slightly lower thermal melting points compared to the non-disease-associated protein RRMs. The full domain of TDP-43 RRM1 and FUS RRM, as well as three β2 peptides from these two RRMs, are prone to fibril formation *in vitro*, and all these fibrils are amyloids in nature. Our results thus confirm, for the first time, by X-ray diffraction and FT-IR analysis that the full domain of RRMs could form amyloids, and contribute to the aggregation-prone properties of TDP-43 and FUS, and could possibly drive protein self-assembly. Thus, our results provide an important basis for further studies of RRMs and RRM-containing proteins in terms of their roles in protein aggregation and neurodegeneration.

## Results

### Most disease-linked RRMs exhibit reversible thermal unfolding and refolding

We expressed 10 His-tagged RRMs from RNA-binding proteins in *E*. *coli*, including 5 RRMs from disease-linked proteins (TDP-43 RRM1, TDP-43 RRM2, FUS RRM, RBM45 RRM1 and RBM45 RRM2), and 5 RRMs from non-disease-associated proteins (U2AF^47^ RRM1, UP1 RRM1, UP1 RRM2, PABP RRM2 and PABP RRM4) (Fig. 1A). These ten RRMs were purified by chromatographic methods to high homogeneity, as revealed by SDS-PAGE (Fig. 1B and Supplementary Fig. S1). To understand the thermal unfolding and refolding processes of these RRMs, we measured their circular dichroism (CD) spectra in the near-UV range (260 to 310 nm) by gradually raising the temperature from 20 °C to 90 °C at intervals of 10 °C, before cooling down from 90 °C to 20 °C, to monitor overall conformational and tertiary structural changes. We noticed that the near-UV CD signal for all of the RRMs was reduced at ~50–70 °C, suggesting that all these RRMs unfolded at high temperatures. Previously, we and others reported the unusual thermal stability of TDP-43 RRM2 using conventional far-UV CD (200 to 260 nm), with this technique providing information on the folding or unfolding of a protein based on secondary structural changes^26,29,44^. Here, using near-UV CD that is more sensitive for detecting overall tertiary structures, we show that the folding of both disease-linked and non-disease-associated RRMs was disrupted at high temperatures. Therefore, diseased-linked RRMs, including TDP-43 RRM2, are not resistant to thermal denaturation based on our near-UV CD data (Fig. 1B).

The refolding process of RRM samples was further monitored by cooling them from 90 °C to 20 °C to assess if the near-UV CD spectra (labeled as “Re-cooled 20 °C” in Fig. 1B) reverted back to the original profiles at low temperatures. We found that most of the disease-linked RRMs— including TDP-43 RRM2, FUS RRM, RBM45 RRM1 and RBM45 RRM2—could be refolded, resulting in CD profiles (displayed as lines of asterisks in Fig. 1B) that closely matched the original profiles measured at low temperatures. TDP-43 RRM1 was the only disease-linked RRM that did not re-fold upon cooling. In contrast, the CD profiles of 3 out of 5 of the non-disease-associated RRMs—including U2AF^47^ RRM1, UP1 RRM1, and UP1 RRM2—did not revert to the original CD profile upon cooling, and the refolded CD profiles of PABP RRM2 and PABP RRM4 only partially matched their original low-temperature profiles (Fig. 1B). In summary, these results show that most of the disease-linked RRMs are not resistant to thermal denaturation as previously reported, but they can be thermally unfolded and this unfolding process is reversible.

### Disease-linked RRMs have a slightly lower average melting point compared to non-disease-associated RRMs

**Figure 2:**
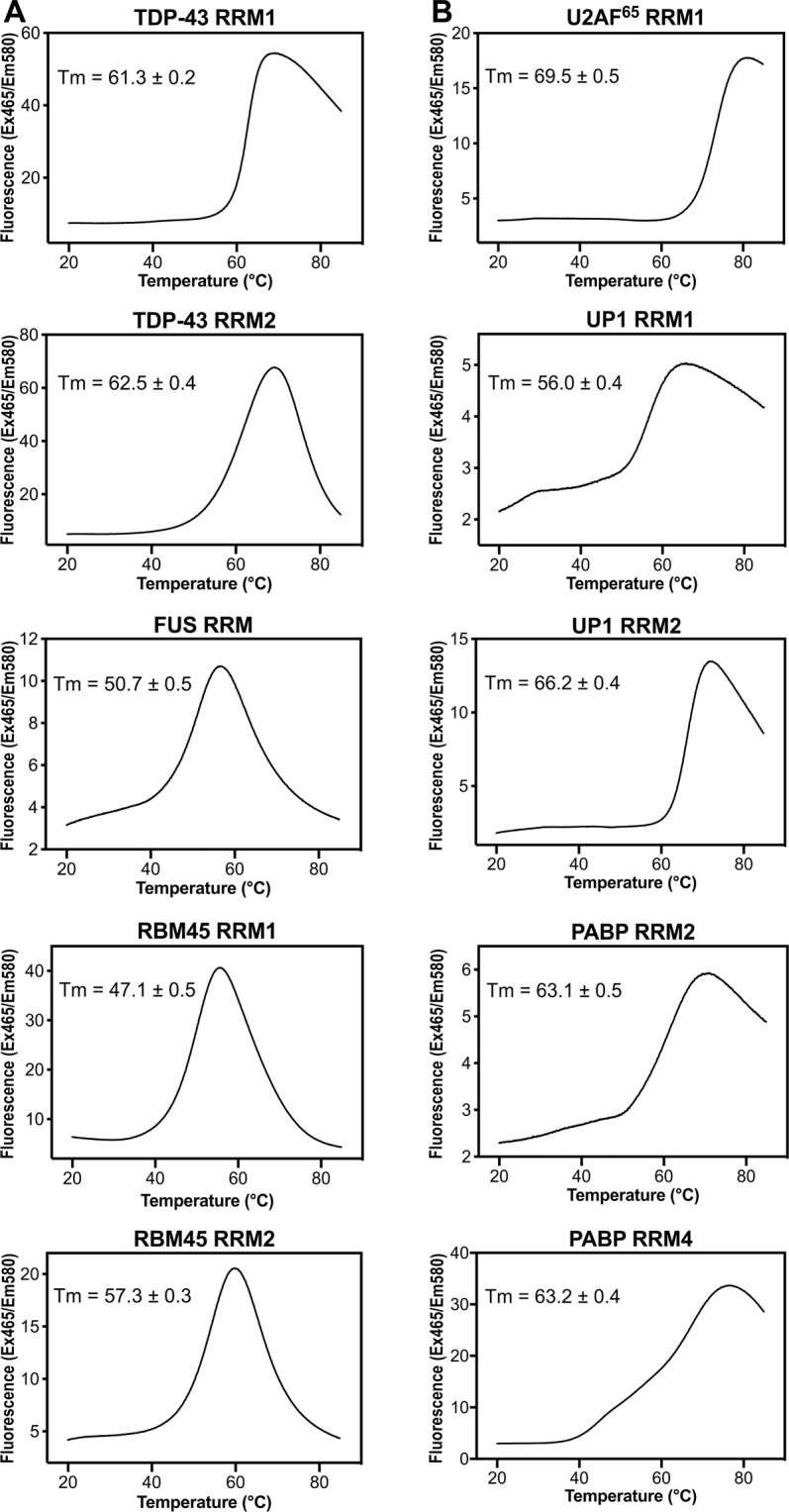
Thermal melting points of disease-linked and non-disease-associated RRMs, measured by differential scanning fluorimetry (DSF). Thermal melting points of RRMs were analyzed by DSF using SYPRO orange dye. The temperature was increased from 20 °C to 85 °C at a rate of 0.06 °C/second, and the emitted SYPRO orange fluorescence signals (excited at 465 nm) were recorded at 580 nm. The thermal melting points (Tm) for (**A**) the disease-linked RRMs (TDP-43 RRM1, TDP-43 RRM2, FUS RRM, RBM45 RRM1, RBM45 RRM2), and for (**B**) non-disease-associated RRMs (U2AF^47^ RRM1, UP1 RRM1, UP1 RRM2, PABP RRM2, PABP RRM4) are shown in each panel.

We next performed differential scanning fluorimetry to measure the thermal melting points of all ten RRMs. We used SYPRO Orange dye as the fluorophore, which binds to unfolded hydrophobic protein surfaces to produce fluorescence signals. We found that RRMs from the aggregation-prone proteins, including TDP-43 RRM2, FUS RRM, RMB45 RRM1 and RBM45 RRM2, shared similar differential scanning fluorimetry profiles, characterized by a sudden decrease in fluorescence signal upon protein melting by raising the temperature (Fig. 2). The five diseased RRMs had slightly lower melting temperatures with an average of ~56 °C (61.3 °C for TDP-43 RRM1, 62.5 °C for TDP-43 RRM2, 50.7 °C for FUS RRM, 47.1 °C for RMB45 RRM1 and 57.3 °C for RBM45 RRM2). The melting points for the non-disease RRMs were slightly higher with an average of ~64 °C (69.5 °C for U2AF^47^ RRM1, 56.0 °C for UP1 RRM1, 66.2 °C for UP1 RRM2, 63.1 °C for PABP RRM2 and 63.2 °C for PABP RRM4). Together with our CD data, these results show that RRMs from disease-linked proteins mostly exhibit reversible thermal unfolding and refolding, and have slightly lower melting points compared to non-disease-associated RRMs.

### FUS RRM and TDP-43 RRM1 form fibril-like aggregates

**Figure 3:**
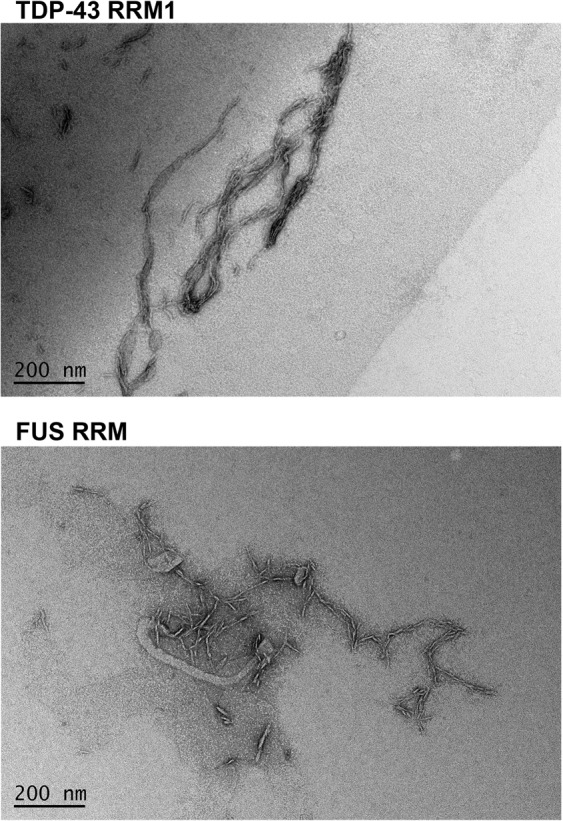
RRMs from TDP-43 and FUS form fibrillar aggregates. Solutions of TDP-43 RRM1 (50 μM) and FUS RRM (50 μM) were agitated in the presence of 10 mM phosphate (pH 7.5) and 50 mM NaCl in room temperature. The freshly-formed fibrillar solutions were examined under negative-stain transmission electron microscopy (EM) to reveal fibril-like aggregates.

We next tested the propensity of the ten RRMs to form fibril aggregates *in vitro*. We found that only TDP-43 RRM1 and FUS RRM could form fibril-like aggregates upon agitation in phosphate buffer at room temperature for two days, i.e., conditions we applied previously to prepare RRM2 peptide fibrils^26^. The fibrillar aggregates of TDP-43 RRM1 and FUS RRM could be viewed by negative-stain transmission electron microscopy (TEM) (Fig. 3). FUS RRM fibrils had a shape similar to the ones reported previously^39^, whereas TDP-43 RRM1 formed long fibril-like structures dissimilar from the RRM1 amorphous aggregates reported before^29^. None of the other eight RRMs we assessed formed fibril-like aggregates under these conditions. However, our data do show that at least two disease-linked RRMs have the unusual capability of self-assembly.

**Figure 4:**
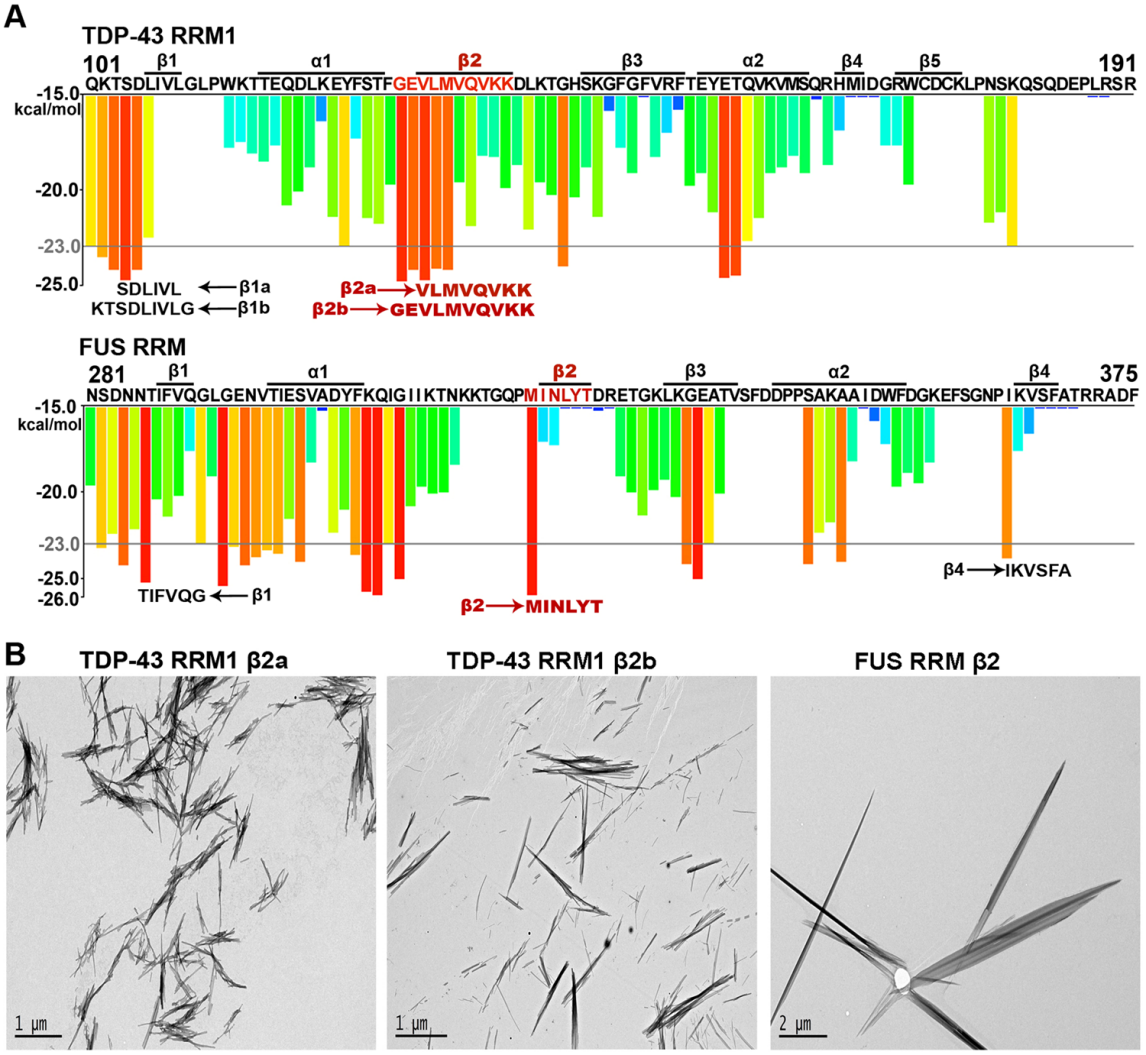
The β2 peptides of TDP-43 RRM1 and FUS RRM are prone to fibril formation. (**A**) Amyloid-promoting segments (predicted by ZipperDB) in TDP-43 RRM1 and FUS RRM are presented as red and orange bars. Amino acid sequences are listed at the top, with secondary structures (labeled as α and β) derived from the crystal structure of TDP-43 RRM1 (PDB entry: 4Y0F) and the NMR structure of FUS RRM (PDB entry: 2LCW) shown above. Three β2 peptides that formed fibrils are labeled in red, whereas the peptides that did not form fibrils are labeled in black, shown at the bottom of each histogram. (**B**) The peptide sequences from the β2 region of TDP-43 RRM1 (β2a and β2b) and FUS RRM (β2) form fibrils *in vitro*, as revealed by negative-stain transmission electron microscopy.

To identify the core aggregation sequences within these two RRMs, we predicted the amyloid-forming segments within TDP-43 RRM1 and FUS RRM using the ZipperDB 3D profiling method^50^. We noticed that a 10-amino acid sequence covering β2 of TDP-43 RRM1 was predicted to have the highest ability to form amyloid fibrils (Fig. 4A). Accordingly, we designed two peptides, named TDP-43 RRM1 β2a (^130^VLMVQVKK^137^) and TDP-43 RRM1 β2b (^128^GEVLMVQVKK^137^). A segment in the β1 region of TDP-43 RRM1 also presented high predictive ability to form amyloid fibrils, so we also designed two peptides in this region, namely β1a (^104^SDLIVL^109^) and β1b (^102^KTSDLIVLG^110^). Three β-strand regions in FUS RRM were also predicted by ZipperDB to be prone to forming amyloids (Fig. 4A), so we designed three respective peptides: β1 (^286^TIFVQG^291^), β2 (^321^MINLYT^326^) and β4 (^364^IKVSFA^369^). Three of these seven peptides formed needle-like fibrils, including TDP-43 RRM1 β2a, TDP-43 RRM1 β2b and FUS RRM β2, which were visualized by negative-stain TEM (Fig. 4B). In summary, our results demonstrate that the β2 strands of TDP-43 RRM1 and FUS RRM may contribute to the fibrillation of RRMs.

### RRM fibrillar aggregates have amyloid properties

**Figure 5:**
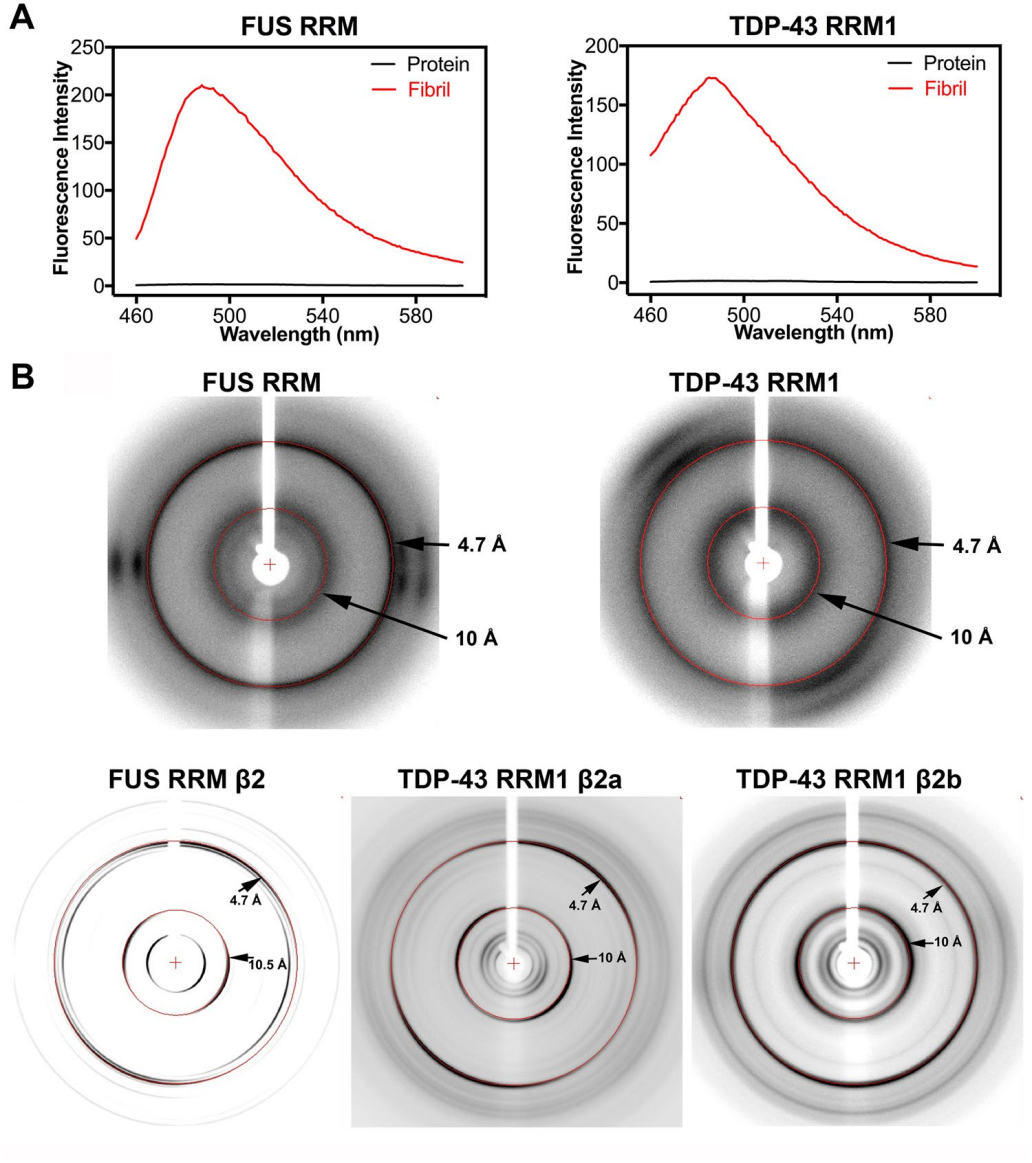
Fibril aggregates of TDP-43 RRM1, FUS RRM and their β2 peptides exhibit amyloid properties. (**A**) Fluorescence spectra (460 to 600 nm, red lines) of the freshly-formed fibrillar solutions of FUS RRM and TDP-43 RRM1 revealed fluorescence signals in the presence of Thioflavin T (excited at 442 nm), but the fresh protein solutions did not generate any signal (black lines). (**B**) X-ray diffraction images of the fibrils formed by FUS RRM, TDP-43 RRM1 and their β2 peptides (TDP-43 RRM1 β2a, TDP-43 RRM1 β2b and FUS RRM β2) reveal the characteristic cross-β diffraction patterns of amyloid fibrils at 4.7 Å and 10 Å (marked by red rings).

To determine if the fibrillar aggregates formed by TDP-43 RRM1 and FUS RRM are amyloids, we incubated these aggregates with the fluorescent dye Thioflavin-T (ThT) that preferentially binds to amyloids and gives a strong fluorescence signal at ~485 nm^51,52^. Fresh protein solutions of FUS RRM or TDP-43 RRM1 did not generate any ThT fluorescence signal. In contrast, aged fibrillar solutions of TDP-43 RRM1 or FUS RRM with ThT generated high fluorescence signal (with maximum emission at 485 nm), suggesting that these fibril-like aggregates exhibit amyloid-like characteristics (Fig. 5A). However, the fibrils formed by β2 peptides of TDP-43 RRM1 and FUS RRM did not generate any fluorescence signal with ThT dye (data not shown), similar to our previous observations for TDP-43 RRM2 β peptides^26^.

To further confirm the amyloid nature of RRM fibrillar aggregates, we used X-ray diffraction to establish if these fibrils could produce the characteristic cross-beta diffraction pattern of amyloids^53–55^. Not only did TDP-43 RRM1 and FUS RRM fibrillar aggregates present the two characteristic diffraction rings—one at 4.7 Å of inter-strand spacing and one at 10 Å of inter-sheet spacing—so did the three β2 peptide fibrils (Fig. 5B and see the summary in Table 1). A previous study showed that a small peptide (^128^GEVLMVQV^135^) from TDP-43 RRM1 could form fibrils *in vitro*, but these fibrils only diffracted X-rays to produce a ring at 9.2 Å, but not the ring at 4.7 Å^31^. Thus, our X-ray diffraction data confirm for the first time that the fibrils formed by TDP-43 RRM1 and FUS RRM (and their respective β2 peptides) are amyloids in nature.

**Table 1:** *In vitro* evidence for amyloid formation by RRMs and peptides within RRMs.

RRM Domain/Peptide Residue numbers		ThT-binding fluorescence signal	Fibrils in EM or AFM	X-ray diffraction cross-β patterns	FTIR	Reference
**TDP-43 RRM1**	101–191	Yes	Yes	Yes	Yes	This study
TDP-43 RRM1 β2a peptide	130–137	No	Yes	Yes	Yes	
TDP-43 RRM1 β2b peptide	128–137	No	Yes	Yes	Yes	
**FUS RRM**	281–375	Yes	Yes	Yes	Yes	
FUS RRM β2 peptide	321–326	No	Yes	Yes	Yes	
TDP-43 RRM2peptide	247–254	Yes	Yes	Yes	NA	ref.^31^
TDP-43 RRM1 peptide	128–135	Not clear	Yes	No	NA	
TDP-43 RRM2 peptides	247–252, 247–255, 247–256, 247–257, 248–253, 248–256, 248–257, 251–259, 252–257 252–258, 253–258	NA	Yes	Yes	NA	ref.^32^
**TDP-43 RRM1**	102–191	Yes	Amorphous aggregates	NA	NA	ref.^29^
**TDP-43 RRM1-2**	102–269	Yes	Amorphous aggregates	NA	NA	
**FUS RRM**	282–371	Yes	Yes	NA	NA	ref.^39^
TDP-43						ref.^26^
Truncated RRM2	208–265	Yes	Yes	NA	NA	
TDP-43 RRM2						
β3 peptides	227–233	No	Yes	NA	NA	
β5 peptide	253–259	No	Yes	NA	NA	

NA: Data not available.

### RRMs and β2 peptide fibrils are amyloids of parallel β-sheets

**Figure 6:**
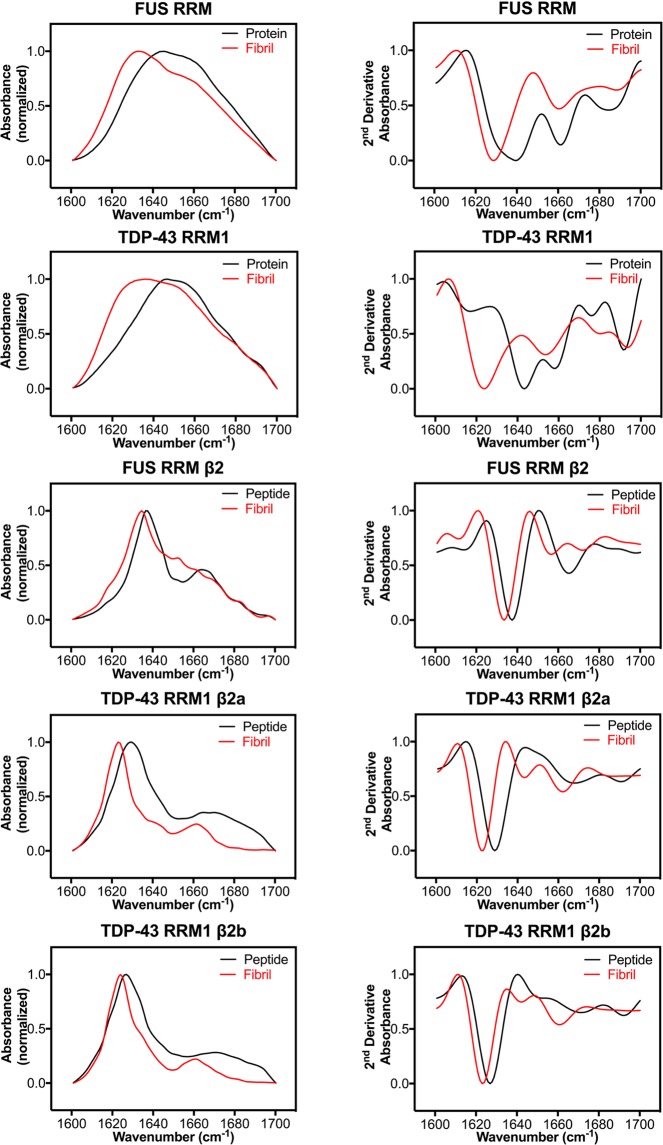
RRM and β peptide fibrils are amyloids of parallel β-sheets based on Fourier transform infrared (FTIR) spectroscopy. The fibrillation properties of FUS RRM, TDP-43 RRM1 and their β2 peptides were analyzed by attenuated total reflection-Fourier transform infrared (ATR-FTIR). ATR-FTIR spectra in the amide I region (1600 to 1700 cm^−1^) are shown in the left column, and the second derivative ATR-FTIR spectra are displayed in the right column. In these ATR-FTIR spectra, the characteristic shift in β-sheet absorbance at 1630–1640 cm^−1^ for fresh protein/peptide (in black) to 1620–1630 cm^−1^ in amyloid fibrils (in red) represents planer extended β-sheet assembly. In the second derivative ATR-FTIR spectra, none of the fibril spectra exhibit a high frequency peak at 1685–1695 cm^−1^, suggesting the presence of amyloid structures consisting of parallel β-sheets.

To further examine the structure of these amyloid-like fibrils, we conducted attenuated total reflection-Fourier transform infrared (ATR-FTIR) spectroscopy; a widely used technique to study the fibrillar conformational changes of β-sheet-rich amyloids^56–59^. We acquired ATR-FTIR spectra for the fresh proteins, fresh peptides, and all fibrils. The ATR-FTIR spectra of the fresh β2 peptides gave the highest absorbance intensities (mainly at 1630–1640 cm^−1^), revealing a characteristic β-strand structure, whereas the spectra for the fresh FUS RRM and TDP-43 RRM1 were broad with a wide frequency range reflecting different secondary structure components (Fig. 6). Comparing the spectra of the fresh TDP-43 RRM1 and FUS RRM with their respective fibrils, we observed a clear peak shift to 1620–1640 cm^−1^ for the fibrils, indicating a conformational change to amyloid β-sheets (see Fig. 6 and Table 1).

To more thoroughly investigate hidden local peaks in the overall spectrum, we calculated second derivatives of each spectrum^40,60^. In doing so, we observed troughs that clearly shifted from 1630–1640 cm^−1^ for fresh proteins/peptides to 1620–1630 cm^−1^ for the aggregates/fibrils (right column in Fig. 6). These 1620–1630 cm^−1^ frequency shifts are characteristic of amyloid fibrils^56,58^, suggesting extended and planar β-sheet formation. Moreover, high-frequency bands at 1685–1695 cm^−1^ disappeared from the spectra of various fibrils (red lines) when compared to the spectra of fresh RRM proteins and peptides (black lines). This type of transition is a characteristic representation of amyloid assembly, indicating that antiparallel β sheets are being transformed into parallel β sheets in proteins and peptide fibrils^56,57,59^. Previous study has also revealed that TDP-43 RRM2 peptides form amyloids of parallel β-sheets^32^, which is in agreement with the parallel amyloid structures observed here for TDP-43 RRM1, FUS RRM and their peptides. In summary, all of our results, including from ThT fluorescence assays, X-ray diffraction and ATR-FTIR spectroscopy, confirm that TDP-43 RRM1, FUS RRM and their β2 peptides can self-assemble into fibril-like amyloids consisting of parallel β-sheets.

## Discussion

Previous studies revealed that TDP-43 RRMs are present in the pathological aggregates in ALS brains by mass spectrometry, suggesting that RRM may contribute to protein aggregation^23^. In this study, we compare the biophysical properties of ten RRMs of disease-related and non-disease-associated RNA-binding proteins to reveal if the disease-linked RRMs share any particular features making them prone to self-assembly. Unexpectedly, we found that the disease-linked RRMs are not resistant to thermal denaturation as suggested previously^26,29,44^, and their tertiary structures could be disrupted by raising the temperature to ~56 °C, as revealed by near-UV CD. This thermal unfolding process is reversible for most of the disease-linked RRMs (except TDP-43 RRM1), as the unfolded RRMs could be refolded upon cooling. In contrast, thermal denaturation of most of the non-disease-associated RRMs is not reversible and these RRMs are mostly precipitated upon heating. We noted that TDP-43 and FUS have previously been shown to undergo liquid-liquid phase separation (LLPS) and form reversible dynamic assemblies mediated by their low complexity C-terminal domains^61–63^. The unique property of reversible thermal unfolding and refolding of disease-linked RRMs supports their conformational flexibility and reversibility, which may induce the intrinsically unfolded C-terminal domains to undergo phase transition or to drive pathological aggregation.

In this study, we also show by X-ray diffraction and ATR-FTIR spectroscopy that TDP-43 RRM1, FUS RRM and their β2 peptides form fibrils and fibril-like aggregates that are amyloids in nature (see summary in Table 1). Previous studies of ALS and FTLD patient tissues reported that TDP-43 cytoplasmic inclusions did not bind amyloid dyes and did not present much fibril-like structure, suggesting they were non-amyloid in nature^47,64,65^. TDP-43 aggregates expressed in yeast cells are not amyloid-like^27^, and TDP-43 purified from inclusion bodies in bacterial cells were structurally amorphous and also non-amyloid^66^. In contrast, fine structural analysis of neuronal inclusions using high-resolution immuno-gold labelling with electron microscopy provided evidence that amyloid-like fibrils are formed by TDP-43 in the affected neurons of patients with neurodegenerative disease^67,68^. Several studies have further reported that the fibrils formed by different regions of TDP-43 are amyloids^14–20,31,32,69^. Recently, it has also been shown by Thioflavin S-staining that TDP-43 forms amyloid-positive aggregates in ALS patients^70^, and the cytoplasmic inclusions in most cases of ALS/FTLD were found to be Thioflavin S-positive upon modifying the staining protocol to remove lipid autofluorescence background^71^. These studies provide strong evidence that protein aggregates in the cytoplasmic inclusions of ALS/FTLD have amyloid-like properties. Our results herein also support that protein aggregates formed by TDP-43 RRM1 and FUS RRMs are amyloids in nature.

Our results also show that all these fibrils, from full-length RRMs or parts thereof, are amyloids with parallel β-sheet structures, similar to the reported structures of the amyloid fibrils Aβ_1–42_ and α-synuclein^72,73^. Hence, our *in vitro*-assembled fibrils from RRMs have structures similar to the pathogenic fibrils in the cytoplasmic inclusions of ALS and FTLD. Moreover, all of the RRM β-peptide fibrils prepared in this study (shown in Fig. 4B) did not produce ThT-binding fluorescence signal. Similar results were reported previously showing that some amyloid fibrils from Aβ_42_ peptides and TDP-43 peptides did not generate ThT-binding signal^15,74^. Despite lack of ThT-binding signal, other lines of evidence presented here (see summary in Table 1), including the EM images, amyloid specific cross-β patterns in X-ray diffraction, and FTIR spectra, show that all these RRM peptide fibrils are amyloids in nature. Our data, together with previous studies, thus provide strong evidence that the ALS and FTLD disease proteins TDP-43 and FUS can form amyloid-like fibrils of pathological relevance. Our data also show, for the first time, that the full domain of RRMs alone can self-assemble into amyloid fibrils by X-ray diffraction and FT-IR analysis, suggesting that RRMs may play a key role in amyloidgenesis.

Overall, our study shows that RRMs of TDP-43, FUS and RBM45 share some unusual properties that are absent from other RRMs, including reversible thermal denaturation and being prone to amyloid formation. Moreover, the parallel β-sheet amyloids formed by TDP-43 RRM1, FUS RRM and their β2 peptides resemble the pathogenic fibrils in degenerating neurons of ALS/FTLD patients. Thus, this study reveals a new avenue for investigating the role of RRMs in amyloid formation and for seeking RRM amyloid-based treatments for neurodegenerative diseases.

## Methods

### Cloning, protein expression and purification

The cDNA of each RRM from the human cDNA library was amplified using 2x PfuUltra II Hotstart PCR Master Mix (Agilent Technologies) and cloned into bacterial expression vector PQE30 or pET28a to generate N-terminal His-tagged recombinant protein-expressing clones. The expression plasmids were transformed into *Escherichia coli* M15 strain or *E*. *coli* BL21-CodonPlus (DE3) RIPL cells, and then incubated at 37 °C overnight in LB media supplemented with 100 μg/mL ampicillin or 40 μg/mL kanamycin. A single bacterial colony was used to inoculate a small 25 ml culture, which was grown overnight. An aliquot (10 ml) was added to 1 L of LB media with antibiotic and this was grown at 37 °C for 3–4 hours. When the optical density of culture at 600 nm (OD_600_) reached 0.6, it was cooled down and induced by adding 0.8 mM isopropyl 1-thio-β-D-galactopyranoside (IPTG). The induced bacterial culture was grown at 18 °C for 16–18 hours for protein overexpression. The cells were harvested by pelleting down the culture and were resuspended in 50 mM phosphate buffer (pH 7.5), 500 mM NaCl and 10 mM β-mercaptoethanol (βME) containing a tablet of cOmplete EDTA-free protease inhibitor cocktail (Roche). The cells were lysed by a microfluidizer (Microfluidics M-110P). The cell lysate was pelleted down by high-speed centrifugation and the supernatant was applied to a HisTrap FF column (GE HealthCare). The His-tagged recombinant proteins were eluted by using an imidazole gradient from 0 to 500 mM. The eluted proteins were further purified by HiTrap Heparin HP columns (GE HealthCare) and loaded into Superdex 75 10/300 GL columns (GE HealthCare) for a final round of purification. Purified protein samples were eluted in a buffer containing 50 mM phosphate buffer (pH 7.5), 150 mM NaCl and 10 mM βME for further experiments. The proteins assessed were: TDP-43 RRM1 (residues 101–191), TDP-43 RRM2 (residues 192–265), FUS RRM (residues 281–375), RBM45 RRM1 (residues 24–109), RBM45 RRM2 (residues 116–197), U2AF^47^ RRM1 (residues 148–237), UP1 RRM1 (residues 7–92), UP1 RRM2 (residues 99–182), PABP RRM2 (residues 95–178), and PABP RRM4 (residues 290–373).

### Circular dichroism spectroscopy

The near-UV CD spectrum of each protein sample was measured using a Chirascan-plus CD spectrometer (Applied Photophysics). To record the near-UV CD signal, each RRM was assessed at a concentration of approximately 2 mg/ml in a buffer of 50 mM phosphate (pH 7.5), 150 mM NaCl and 0.5 mM Tris(2-carboxyethyl)phosphine (TCEP). The spectra were recorded in the near-UV range (from 260 nm to 310 nm) in a 10-mm path length quartz cuvette at temperatures increasing from 20 °C to 90 °C at 10-degree intervals with an equilibration time of 3 minutes. The samples were then slowly cooled down to 20 °C within 30 minutes and the spectra were recorded again to monitor the structures upon re-cooling.

### Differential scanning fluorimetry

The thermal melting curve of each RRM was measured in triplicate by differential scanning fluorimetry using a LightCycler 480 system (Roche). A final protein concentration of 10 μM and 15X SYPRO Orange dye (Invitrogen) was mixed in each well in a LightCycler Multi-well Plate 96 White (Roche). The temperature was raised from 20 °C to 85 °C at a rate of 0.06 °C/second with 10 acquisitions per degree. An excitation filter of 465 nm and an emission filter of 580 nm were used for SYPRO Orange detection. Melting temperatures (Tm) were calculated by LightCycler^®^ Protein Melting Analysis.

### *In vitro* fibril formation and EM imaging

The purified sample of each RRM was diluted into a buffer containing 10 mM phosphate (pH7.5) and 50 mM NaCl to a final concentration of 50 μM. RRM samples were then centrifuged at 20,000 *g* for 10 minutes before being filtered through a 0.22 µm Millex-GV filter (Millipore) to remove any insoluble material or aggregates. All β2-peptides (synthesized by Mission Biotech) of TDP-43 RRM1 and FUS RRM were dissolved in water, and both RRM and peptide samples were agitated at room temperature for two days to promote fibril formation. The freshly-formed fibrillar solutions (5 μL) were placed on 200-square-mesh carbon-coated, glow-discharged grids (Electron Microscopy Science). Each grid was washed with water and stained by 1% uranyl acetate for 2 minutes at room temperature and then air-dried. Fibrils on the grids were examined by a Tecnai G2 Spirit TWIN transmission electron microscope (FEI Company).

### Thioflavin T (ThT) binding assays

Thioflavin T (ThT, Sigma) was dissolved in water to make a stock solution (1 mM), which was filtered through a 0.22 μm filter and stored at −20 °C with protection from light. This ThT stock was diluted in 10 mM phosphate buffer to a concentration of 25 μM. Fresh protein or fibrillar solutions (100 μL) were mixed with 400 μL ThT dye (to a final concentration of 20 μM) and incubated for 5 minutes in the dark. The samples were excited at 442 nm, and the fluorescence emission signal was recorded from 460 to 600 nm using a Varian Cary Eclipse Fluorescence Spectrophotometer (Agilent) in a 400-μL fluorescence micro cell (Varian).

### X-ray diffraction

The freshly made fibrillar solution of peptides/RRMs were centrifuged at 20,000 g for 60 minutes and washed with Milli-Q water. The dried fibrils were placed on the CrystalCap CryoLoop. X-ray diffraction images were recorded at room temperature using Rigaku Ultimate HomeLab protein crystallography system equipped with Saturn 944+ CCD detector and FR-E+ SuperBright microfocus rotating anode generator.

### Fourier-transform infrared (FTIR) measurements

The structural transition from proteins/peptides to fibrils was studied by attenuated total reflection FTIR (ATR-FTIR). The ATR-FTIR spectra were recorded on a Tensor 27 FTIR spectrophotometer (Bruker) in conjunction with OPUS data collection software. We used high concentrations (~12 mg/ml) of fresh protein samples for FTIR analysis. The freshly prepared fibrils were washed three times with D_2_O water to remove any residual proteins and were then re-suspended in a small amount of D_2_O. The samples were evenly spread on an internal reflection element (IRE) crystal using a micropipette tip. The buffer and D_2_O spectra were used as background for protein and fibril samples, respectively. The data were collected as an average of 128 scans at 1 cm^−1^ resolution. PeakFit (Systat Software Inc.) was used for spectral processing and data analysis. We plotted the amide I band at 1600–1700 cm^−1^ to record specific spectral features in amyloids.

## Supplementary information

Supplementary Information
