# Supplementary material for: RNA recognition motifs of disease-linked RNA-binding proteins contribute to amyloid formation
Source: Sci Rep. 2019 Apr 16;9:6171. doi: 10.1038/s41598-019-42367-8 (PMC6467989; doi:10.1038/s41598-019-42367-8)
Supplement: Supplementary file 1 — Supplementary Information [file 41598_2019_42367_MOESM1_ESM.pdf]

Supplementary Information

RNA recognition motifs of disease-linked RNA-binding proteins contribute to amyloid formation

Sashank Agrawal[1,2], Pan-Hsien Kuo[2], Lee-Ya Chu[2,3,4], Bagher Golzarroshan[2],
Monika Jain[1,2] and Hanna S. Yuan[1,2*]

This file includes:

Supplementary Figure 1

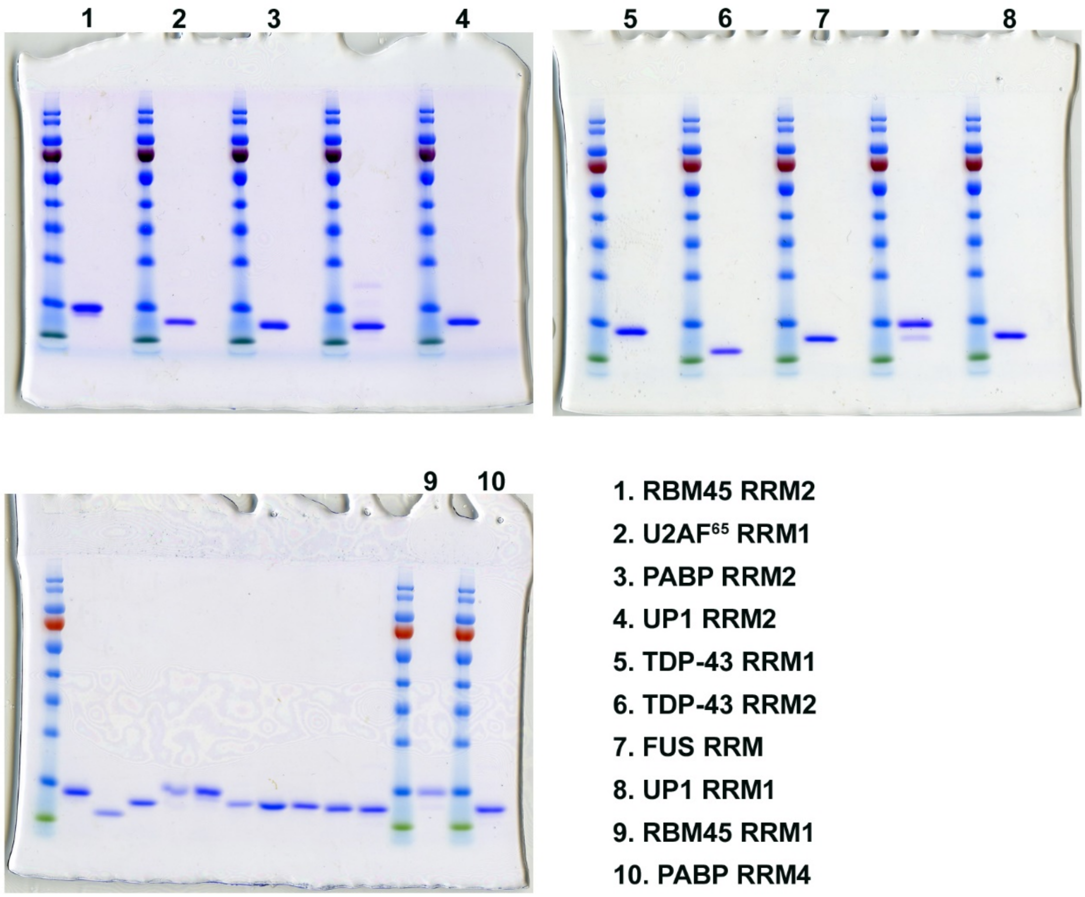

Supplementary Figure 1. The full-length SDS-PAGE gels show that each of the ten RRMs was purified to a high homogeneity (cropped images are shown in Figure 1).
